# The Influencing Factors of In Vitro Regeneration and Bulblet Enlargement of Two Ploidy *Lilium longiflorum*

**DOI:** 10.3390/plants15091356

**Published:** 2026-04-29

**Authors:** Ningya Chen, Xiaodan Wu, Ke Wang, Yu Ren, Zongyang Jin, Guixia Jia

**Affiliations:** Beijing Key Laboratory of Ornamental Plants Germplasm Innovation & Molecular Breeding, National Engineering Research Center for Floriculture, Beijing Laboratory of Urban and Rural Ecological Environment, Key Laboratory of Genetics and Breeding in Forest Trees and Ornamental Plants of Ministry of Education, School of Landscape Architecture, Beijing Forestry University, Beijing 100083, China

**Keywords:** *Lilium longiflorum*, scale differentiation, bulblet enlargement, tissue culture, RT-qPCR

## Abstract

*Lilium longiflorum* is a diploid lily species valued for its tolerance to humid–hot environments and pleasant fragrance. However, its poor cold hardiness and low bulb-forming capacity limit its cultivation. To overcome these deficiencies, autotetraploids were previously generated in our laboratory via somatic doubling. In order to expand the reproductive efficiency of the two, this study optimized in vitro regeneration and bulblet enlargement protocols. We analyzed the effects of various plant growth regulators and sucrose concentrations, alongside the expression of genes related to carbohydrate metabolism and hormone signaling. Results revealed divergent regenerative pathways: diploids favored direct organogenesis (optimal medium: MS + 30 g/L sucrose + 0.5 mg/L 6-BA + 0.2 mg/L NAA + 1.0 mg/L glyphosate), whereas tetraploids thrived via a TDZ-induced callus pathway (1/2 MS + 30 g/L sucrose + 1.0 mg/L NAA + 0.2 mg/L TDZ). During bulblet enlargement, diploids were predominantly regulated by IBA and prone to proliferation (optimal enlargement medium: MS + 60 g/L sucrose + 2.0 mg/L IBA), while tetraploids were sucrose-sensitive and prioritized single-bulb hypertrophy (MS + 60 g/L sucrose + 0.5 mg/L IBA + 0.1 mg/L 6-BA + 0.1 mg/L CPPU). qRT-PCR indicated that *LlAGPS1*, *LlGBSSI*, *LlSWEET15*, *LlMYC2*, and *LlSAUR32* were highly expressed in tetraploids during rapid enlargement (24–36 d), suggesting a role in bulb hypertrophy, whereas upregulated *LlSUS4* and *LlCWIN3* in diploids correlated with proliferation. The study provides a practical technical reference for the industrialized propagation of high-quality *L.longiflorum* bulbs and provide a theoretical foundation for understanding ploidy-dependent development in Lilium.

## 1. Introduction

*Lilium longiflorum* is a perennial herbaceous bulbous plant belonging to the family Liliaceae and is native to Taiwan, China, and the Ryukyu Islands [1]. Its flowers are pure white and trumpet-shaped, with an elegant morphology, conferring high value in both ornamental horticulture and commercial production. However, its relatively poor cold tolerance and difficulty in bulb formation lead to increased production costs in colder regions. Polyploidization has emerged as a transformative breeding strategy to overcome such limitations, as genome doubling often confers enhanced environmental resilience, increased vigor, and a broader ecological niche compared to diploid progenitors [2]. Moreover, Polyploidization often triggers metabolic changing, notably through the modulation of primary metabolite profiles that underpin enhanced growth and developmental plasticity [3]. Therefore, tetraploid *L. longiflorum* was previously obtained in this study through somatic chromosome doubling. To exploit the genetic potential of polyploids, in vitro regeneration serves as an indispensable means for rapid propagation and physiological evaluation. In vitro culture is an efficient and high-quality method for propagating bulbs of original strains and virus-free seedlings. A conventional approach involves the induction of adventitious shoots from bulb scales, followed by specialized cultivation pathways to promote bulblet formation. The size of these in vitro bulblets is a critical determinant of post-transplant survival, with larger bulblets consistently exhibiting higher establishment rates. However, the optimal formulations of plant growth regulators (PGRs) for scale differentiation and subsequent enlargement vary markedly across Lilium genotypes. Therefore, it is imperative to develop tailored, high-efficiency protocols that account for the specific requirements of different genetic backgrounds.

The differentiation and development of in vitro lily bulblets are synergistically regulated by various endogenous and exogenous factors, notably plant growth regulators (PGRs) and carbohydrate supply. Sucrose functions not only as an essential carbon and energy source but also as a signaling molecule that drives bulblet hypertrophy by stimulating cell division [4]. Furthermore, the specific types, concentrations, and stoichiometric ratios of PGRs are decisive in governing the direction of organogenesis; historically, the combination of 6-BA with auxins such as NAA or IBA has remained the conventional formulation. Recently, novel regulators—including thidiazuron (TDZ), glyphosate, and forchlorfenuron (CPPU)—have demonstrated significant potential in the micropropagation of ornamental species. For instance, TDZ-supplemented media are instrumental in promoting adventitious shoot proliferation [5,6], while low concentrations of glyphosate have been shown to significantly enhance shoot induction frequency in “Jinghe” lily [7]. Additionally, CPPU has proven effective in modulating the expansion of storage organs, including fruits, tubers, and bulbs [8]. However, the specific effects of these regulators on in vitro bulblet differentiation and enlargement in diploid and tetraploid *L. longiflorum* remain to be further elucidated.

At the mechanistic level, the differentiation and enlargement of lily bulbs involve a series of carbohydrate regulatory processes, particularly the synthesis of starch and soluble sugars and the activities of their associated enzymes. The metabolism and transport of these carbohydrates contribute directly to organogenesis and development. Previous studies have indicated that the initiation of bulb enlargement in lilies is marked by a concurrent increase in both starch and soluble sugar contents [9]. To further investigate the molecular underpinnings of these processes, key candidate genes were systematically identified based on their essential roles within the carbohydrate metabolic and transport pathways. Among these, as the predominant storage carbohydrate, starch sequestration is pivotal for nutrient partitioning and morphological bulb development. This process is coordinately regulated by key enzymes and their encoding genes, such as ADP-glucose pyrophosphorylase (AGPase) and granule-bound starch synthase (GBSS), whose expression levels correlate positively with starch accumulation and the magnitude of enlargement [10,11]. In addition, sucrose, as the predominant soluble sugar, serves as a central nutrient carrier during lily bulb development, directly participating in metabolic activities and long-distance transport. During sugar metabolism, sugar will eventually be exported transporters (SWEETs) play important roles in apoplastic sucrose loading and unloading from source to sink tissues, thereby influencing soluble sugar accumulation and the development of storage organs [12,13]. Similarly, sucrose synthase (SUS) is involved in sucrose cleavage and represents a key enzyme affecting the number and size of underground storage organs [14,15], while cell wall invertase (CWIN) catalyzes the hydrolysis of sucrose into hexoses, essential for the differentiation of sink tissues [16]. Regarding phytohormonal orchestration, auxin modulates bulblet expansion by regulating downstream signaling cascades, including the *SAUR* gene family, which facilitates cell elongation [9,17]. In addition, jasmonic acid and its central transcription factor *MYC2* also play important roles in bulb enlargement [18]. Despite the established roles of these carbohydrate metabolic and hormonal pathways in various plant species, the comparative regulatory patterns governing in vitro bulblet enlargement in diploid versus tetraploid *L. longiflorum* remain largely unexplored.

To facilitate the efficient large-scale production and commercial cultivation of *L. longiflorum*, this study aims to systematically establish and optimize in vitro protocols tailored to the distinct physiological requirements of diploid and tetraploid genotypes. Using scale-derived explants, we evaluated the synergistic effects of various plant growth regulators and sucrose concentrations to identify the most effective media for adventitious shoot induction and bulblet enlargement. Furthermore, the expression dynamics of key genes—including those involved in carbohydrate metabolic (*LlGBSSI*, *LlAGPS1*, *LlSWEET15*, *LlSUS4*, *LlCWIN3*) and hormone signaling (*LlSAUR32*, *LlMYC2*)—were analyzed via RT-qPCR to provide molecular validation for the observed growth patterns. Ultimately, these findings offer a practical technical reference for the industrialized propagation of high-quality lily bulbs and provide a theoretical foundation for understanding ploidy-dependent development in Lilium.

## 2. Results

### 2.1. Chromosome Ploidy Observation of Diploid and Tetraploid L. longiflorum

**Figure 1:**
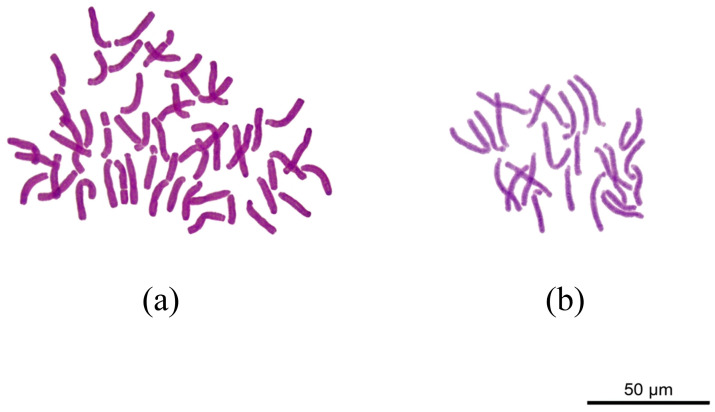
Chromosome ploidy of tetraploid and diploid *L. longiflorum.* (**a**) 4 L. (**b**) 2 L.

Root tips from 10 randomly selected culture bottles were used for chromosome ploidy identification. Among the 20 plantlets labeled as 4 L, all individuals consistently exhibited a chromosome number of 48 (Figure 1a), confirming their status as stable tetraploids (the haploid number n = 12 for *L. longiflorum*). For the 20 plantlets labeled as 2 L, cytological observation revealed that all individuals possessed a stable chromosome count of 24 (Figure 1b). The ploidy levels of both 4 L and 2 L plantlets were highly stable, making them suitable for subsequent experiments. In the following figures and tables ‘4 L’ and ‘2 L’ denote tetraploid and diploid *L. longiflorum*, respectively.

### 2.2. Effects of Different Induction Media on the Differentiation of Diploid and Tetraploid L. longiflorum Bulb Scales

#### 2.2.1. Effects of CPPU Combined with 6-BA or NAA on Adventitious Shoot Differentiation from Bulb Scales of Diploid and Tetraploid *L. longiflorum*

Under different combinations of CPPU with 6-BA or NAA, bulb scales of both diploid and tetraploid *L. longiflorum* regenerated exclusively via adventitious shoot formation, with overall shoot induction frequency approaching 1, indicating that the addition of CPPU does not alter the differentiation pathway of the scales. Regarding the differentiation coefficient, media combining CPPU with NAA induced significantly more adventitious shoots than media combining CPPU with 6-BA (*p* < 0.05), with slightly earlier shoot emergence and faster growth rates, suggesting that CPPU in combination with NAA is more favorable for adventitious shoot induction from *L. longiflorum* bulb scales. Compared with the conventional medium (MS + 0.5 mg/L 6-BA + 0.2 mg/L NAA), the addition of CPPU did not significantly increase the differentiation coefficient in diploid materials, indicating that CPPU has limited effect on adventitious shoot induction in diploid scales. In contrast, in tetraploid materials—particularly under the CPPU + NAA combination—the differentiation coefficient was significantly higher than that in the conventional medium (MS + 0.5 mg/L 6-BA + 0.2 mg/L NAA) (*p* < 0.05), demonstrating that CPPU more effectively enhances in vitro regeneration and proliferation in tetraploid *L. longiflorum* (Table 1).

#### 2.2.2. Effects of Glyphosate on Adventitious Shoot Differentiation from Bulb Scales

After the addition of glyphosate to media containing 6-BA and NAA, the differentiation pathway of the bulb scales remained unchanged, with regeneration occurring exclusively via adventitious shoot formation. The shoot induction frequency of explants exhibited high stability across both ploidy levels and all glyphosate concentrations, consistently remaining near 1.0. This indicates that the initiation of adventitious shoots was not sensitive to the tested concentration range, with the observed experimental variations primarily manifesting in the subsequent differentiation coefficients. Differences were observed between the two ploidy levels of *L. longiflorum* in terms of germination time, shoot induction frequency, and differentiation coefficient. Overall, tetraploid *L. longiflorum* exhibited slightly earlier shoot emergence than diploid plants, whereas both the shoot induction frequency and differentiation coefficient were lower than those of the diploid. In tetraploid materials, the differentiation coefficient increased with increasing glyphosate concentration and reached a maximum (2.88) at 3.0 mg/L; however, partial browning and death of some scales were observed at this concentration. In diploid materials, the addition of glyphosate significantly enhanced the differentiation coefficient compared with the control group (supplemented with 0 mg/L TDZ); Nevertheless, as glyphosate concentration increased from 1.0 mg/L to 3.0 mg/L, the differentiation coefficient showed a clear decreasing trend (from 3.64 to 3.08 and 3.26). The highest differentiation coefficient (3.64) was obtained at 1.0 mg/L glyphosate, under which no scale browning was observed (Table 2). Therefore, the most suitable medium for the induction and differentiation of diploid *L. longiflorum* bulb scales was MS + 30 g/L sucrose + 0.5 mg/L 6-BA + 0.2 mg/L NAA + 1.0 mg/L glyphosate.

#### 2.2.3. Effects of 1/2 MS Medium Supplemented with NAA and TDZ on Adventitious Shoot Differentiation

**Figure 2:**
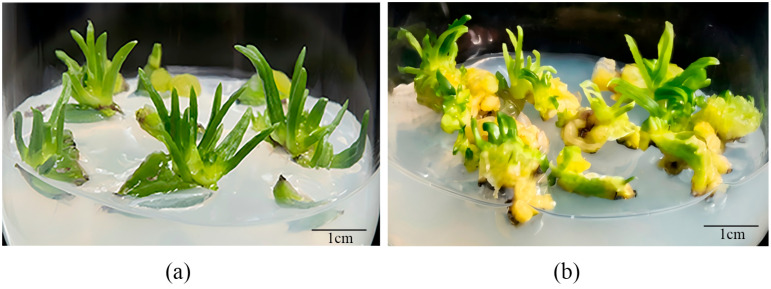
The differentiation pathway of scales changed after adding different concentrations of TDZ. (**a**) The differentiation pathway of scales without TDZ was adventitious buds; (**b**) The differentiation pathway after adding different concentrations of TDZ was callus.

Compared with the control, the addition of different concentrations of TDZ redirected bulb scale differentiation toward a callus-mediated pathway, followed by the formation of adventitious shoots (Figure 2). Under TDZ treatment, shoot emergence occurred earlier in tetraploid *L. longiflorum* but was delayed in diploid plants. When TDZ was applied at 0.2 mg/L, both ploidy levels of *L. longiflorum* exhibited relatively favorable differentiation responses. Moreover, at all tested TDZ concentrations, tetraploid materials showed higher differentiation coefficients than diploid ones (Table 3). Notably, in the present study, the treatment 1/2 MS + 30 g/L sucrose + 1.0 mg/L NAA + 0.2 mg/L TDZ resulted in the highest differentiation coefficient among all 14 bulb scale induction combinations (including all the induction combinations mentioned in Section 2.2.1, Section 2.2.2 and Section 2.2.3) for tetraploid *L. longiflorum*, and also promoted earlier shoot emergence. These results (Table 1, Table 2 and Table 3) showed that tetraploid bulb scales achieve higher differentiation coefficients through a callus-mediated pathway.

### 2.3. Combined Effects of IBA, CPPU, 6-BA, and Sucrose on Bulblet Enlargement in Diploid and Tetraploid L. longiflorum

A four-factor, three-level orthogonal experimental design was adopted, and the induced bulblets were cultured onto the different culture media described below; the detailed results are presented in Table 4. A Pearson correlation analysis revealed a significant positive correlation between bulblet diameter and enlargement coefficient (r = 0.92 for 4 L and r = 0.74 for 2 L; *p* < 0.01), confirming the internal consistency of these two growth indicators. Using the bulblet enlargement coefficient as the primary evaluation index, tetraploid *L. longiflorum* exhibited the highest enlargement coefficient (4.08) on medium No. 4, namely MS + 60 g/L sucrose + 0.5 mg/L IBA + 0.1 mg/L6-BA + 0.2 mg/L CPPU. Diploid *L. longiflorum* showed the highest enlargement coefficient (3.24) on medium No. 6, namely MS + 60 g/L sucrose + 2 mg/L IBA + 0.1 mg/L CPPU. Analysis of variance (ANOVA) based on the bulblet enlargement coefficient indicated that all factors exerted extremely significant effects (*p* < 0.01) on bulblet enlargement in tetraploid *L. longiflorum*, with the magnitude of their effects ranked as sucrose > IBA > 6-BA > CPPU based on the F-values (Table 5). Multiple comparison analysis identified the optimal medium combination for tetraploid bulblet enlargement as MS + 60 g/L sucrose + 0.5 mg/L IBA + 0.1 mg/L 6-BA + 0.1 mg/L CPPU. For diploid *L. longiflorum*, sucrose and IBA had extremely significant effects on bulblet enlargement, with IBA showing the greatest influence, whereas differences among concentrations of 6-BA and CPPU were not significant. Multiple comparison analysis indicated that the suitable medium combination for diploid bulblet enlargement was MS + 60 g/L sucrose + 2 mg/L IBA.

### 2.4. Developmental Progression of Bulblet Enlargement and Associated Gene Expression

#### 2.4.1. Bulblet Developmental Process

**Figure 3:**
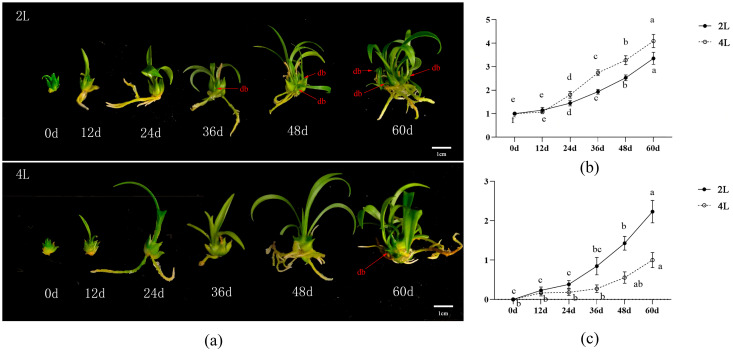
The bulblet development of 2 L and 4 L. (**a**) Morphological changes of 2 L and 4 L bulblets during enlargement development db: daughter bulblets; (**b**) Bulblet enlargement coefficient; (**c**) The number of daughter bulblets. The different lowercase letters in the graph represented significant differences among the data at the *p* < 0.05 level.

After diploid and tetraploid *L. longiflorum* were transferred to the optimal bulblet-enlargement media screened above, the bulblets continued to grow, as evidenced by an increase in leaf number, continuous root elongation, an increase in fresh bulblet weight, and the gradual differentiation of daughter bulblets at the basal part of the main bulblet (Figure 3a). However, clear differences were observed in the developmental progression between diploid and tetraploid plants. In terms of bulblet enlargement, no significant increase in fresh weight was detected in either ploidy during the first 0–12 d of culture. Between 24 and 36 d, the enlargement rate of tetraploid bulblets increased markedly and exceeded that of diploids at the same stage. By the end of culture (60 d), the enlargement coefficients of tetraploid and diploid bulblets reached 4.09 and 3.35 respectively, indicating that tetraploids possess a clear advantage in bulblet enlargement (Figure 3b). During the bulblet enlargement culture, diploid plants tended to produce a greater number of new daughter bulblets at the basal region of the bulblet. After 60 d of culture, the average number of newly differentiated bulblets reached 2.23 per bulblet in diploids, whereas tetraploids produced an average of only 1.00 bulblet (Figure 3c). Taken together, these results indicate that *L. longiflorum* plants with different ploidy levels exhibit distinct growth and developmental tendencies during bulblet enlargement culture. To elucidate the physiological basis underlying these differences, further analyses were conducted focusing on cytological structures as well as the expression of genes related to carbohydrate metabolism and hormone signaling pathways.

#### 2.4.2. Cytological Observation of in Vitro–Cultured Scales

**Figure 4:**
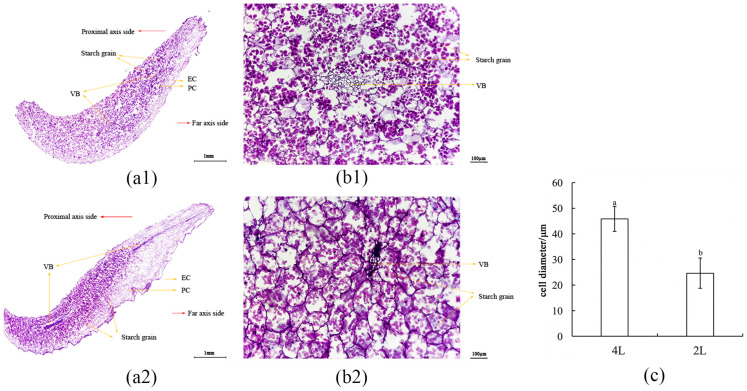
Histological Observation of Scale Cells in Diploid and Tetraploid *L. longiflorum*. (**a1**) Middle longitudinal section of 2 L scale; (**a2**) Middle longitudinal section of 4 L scale; (**b1**) Middle transverse section of 2 L scales; (**b2**) middle transverse section of 4 L scales; (**c**) cell size statistics; PC: Parenchyma cell; EC: Epidermal cells; VB: Vascular bundle tissue. The different lowercase letters in the graph represented significant differences among the data at the *p* < 0.05 level.

For cytological observation, transverse sections were prepared from the scales of diploid and tetraploid *L. longiflorum* bulblets following a 60-day culture period. Longitudinal sections of the scales (Figure 4(a1,a2)) showed that starch granules were mainly concentrated in the middle and basal regions on the adaxial side of the scales. Statistical analysis of parenchyma cell size in transverse sections from the middle region of the scales (Figure 4(b1,b2)) indicated that cells of tetraploid *L. longiflorum* were significantly larger than those of diploids (Figure 4c). The parenchyma cells contained abundant starch granules, predominantly as single granules. Compared with diploids, starch granules in tetraploid cells were more densely accumulated. Clearly defined vascular bundles were also observed, within which purple-red soluble sugars and related substances were present. In diploids, starch was relatively evenly distributed in cells surrounding the vascular bundles, whereas in tetraploids, starch granules were densely accumulated in cells adjacent to the vascular bundles. Transmission electron microscopy further revealed that bulblet enlargement during in vitro culture was accompanied by the formation and accumulation of starch granules.

#### 2.4.3. Expression of Related Genes During In Vitro Bulblet Development

During in vitro bulblet enlargement culture of diploid and tetraploid *L. longiflorum*, the expression of seven genes related to bulblet enlargement and proliferation, involving carbohydrate metabolism and hormone metabolism was analyzed. Quantitative real-time PCR results showed that the expression patterns of these seven genes during bulblet development could be classified into two distinct trends.

**Figure 5:**
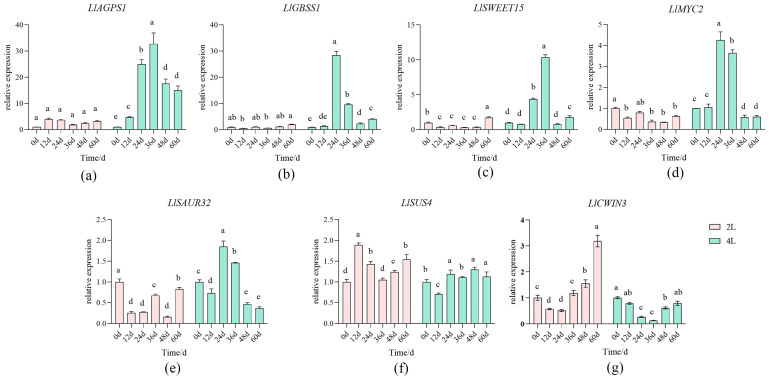
Relative expression levels of associated genes during in vitro bulb enlargement in two ploidy *L. longiflorum.* (**a**) Expression level of *LlAGPS1*; (**b**) Expression level of *LlGBSSI*; (**c**) Expression level of *LlSWEET15*; (**d**) Expression level of *LlMYC2*; (**e**) Expression level of *LlSAUR32*; (**f**) Expression level of *LlSUS4*; (**g**) Expression level of *LlCWIN3*; Note: Different lowercase letters in the same column represent the difference significance of data at the *p* < 0.05 level.

One trend, including the starch biosynthesis genes *LlAGPS1* and *LlGBSSI,* the sucrose transporter gene *LlSWEET15*, the auxin-responsive gene *LlSAUR32*, and the transcription factor *LlMYC2* (Figure 5a–e) in the jasmonic acid (JA) signaling pathway, maintained consistently low expression levels in diploid scales, showing only slight fluctuations throughout the 0–60 d developmental period. In contrast, these genes were expressed at relatively high levels in tetraploids and exhibited significant temporal variation during bulblet development: expression levels were low at the early stage of bulblet enlargement (0–12 d), increased markedly at 24–36 d, and then gradually declined thereafter.

Another trend, comprising the sucrose synthase gene *LlSUS4* and the cell wall invertase gene *LlCWIN3* (Figure 5f,g), exhibited overall higher expression levels in diploid bulblets compared with tetraploids during bulblet development. Expression of *LlSUS4* remained relatively stable in both ploidy levels of *L. longiflorum*. *LlCWIN3* showed a similar temporal trend in both diploid and tetraploid bulblets. Specifically, its expression profile exhibited a U-shaped pattern, characterized by a gradual down-regulation from 0 d to 24 d (or 36 d), followed by a subsequent sharp increase in the later stages. But its expression level was notably higher in diploids, particularly after 36 d of culture, where a significant increase was observed.

## 3. Discussion

### 3.1. Differences in Differentiation Pathways and Hormonal Responses Between Diploid and Tetraploid L. longiflorum

In this study, diploid and tetraploid *L. longiflorum* exhibited distinct preferences for optimal regeneration pathways. Diploids achieved the highest differentiation efficiency through direct organogenesis on MS medium supplemented with 0.5 mg/L 6-BA, 0.2 mg/L NAA, and 1.0 mg/L glyphosate. This is consistent with earlier findings that direct adventitious shoot formation is a primary regeneration route for many Lilium species [19,20]. The promotive effect of low-concentration glyphosate in diploids aligns with the observations of Bowe et al. [21], suggesting that such chemical stimuli can modulate endogenous hormone levels, particularly the balance of auxins, to facilitate rapid shoot primordia initiation. For tetraploid bulb scales, although direct regeneration remained feasibility, a significantly higher differentiation coefficient (4.15) was obtained through a callus-mediated pathway using 0.2 mg/L TDZ. As a phenylurea derivative, TDZ is known for its strong cytokinin-like activity, which often promotes cell dedifferentiation and callus induction more effectively than purine-type cytokinins like 6-BA [22]. Our results indicate that while both ploidies retain the capacity for organogenesis, the optimal physiological state for tetraploid regeneration shifts toward a callus-mediated process under TDZ treatment. This divergence supports the view of Levin [23] that polyploidization can alter cellular sensitivity and the required concentration thresholds for exogenous plant growth regulators. The increased efficiency of the callus-mediated route in tetraploids suggests that the reorganized physiological state of polyploid cells may respond more robustly to the intensive stimulus of TDZ, leading to a more efficient transition of cell fate during in vitro culture.

### 3.2. The Combination of Higher Concentrations of Sucrose and Suitable Plant Growth Regulators Facilitates Bulblet Enlargement In Vitro

Sucrose plays a dual role in *L. longiflorum* bulblet development, serving as both a primary energy source and a signaling molecule that triggers physiological reprogramming [24]. Our results showed that a sucrose concentration of 60 g/L maximized the enlargement coefficient in both ploidies, which aligns with the concentration-dependent response pattern observed in other Lilium species [25,26]. However, when the concentration was increased to 90 g/L, a decline in bulblet enlargement was observed. This inhibitory effect is attributed to the excessive osmotic potential in the medium, which can lead to cellular dehydration and osmotic stress, thereby overriding the growth-promoting effects of high carbon availability [27].

There are various mechanisms by which exogenous plant hormones induce bulblet enlargement, such as the type of hormone, its concentration, and interaction. Storage underground organs are closely associated with cytokinin and auxin. Notably, we observed a ploidy-difference response to cytokinins (6-BA and CPPU). Tetraploid *L. longiflorum* showed enhanced enlargement when these regulators were added, whereas diploids achieved stable growth with IBA alone. This suggests that polyploidization may alter the endogenous hormonal balance or receptor sensitivity. In diploids, the primary growth limitation appears to be root-mediated nutrient absorption [28], which is compensated for by exogenous IBA. Indole-3-butyric acid (IBA), a synthetic plant growth regulator, stimulates cell proliferation and division. Not only does it initiate roots but it also helps develop the root system and enhances photosynthetic performance in plants [29]. The result of this study was that IBA exerted an extremely important effect on bulblet enlargement in both diploid and tetraploid *L. longiflorum*. According to the current report, this finding is in line with prior studies reporting that application of suitable IBA concentration to the culture medium increases the formation and enlargement of the bulblets in lilies [30]. Therefore, IBA demonstrates substantial application potential and value in driving the enlargement of tissue-cultured bulblets of both ploidy *L. longiflorum*.

### 3.3. Physiological and Molecular Differences in the Bulblet Enlargement Process of Diploid and Tetraploid L. longiflorum

It was found in the course of the study that diploid and tetraploid *L. longiflorum* have different approaches to the process of bulblet enlargement: diploids usually grow more daughter bulblets at relatively low rates of bulblet enlargement compared to tetraploids which prefer the rapid growth of individual bulblets. This pattern can be observed in cytology as well as in gene expression studies.

At the cellular level, more dense starch granule accumulation is observed within tetraploid parenchyma cells, particularly around vascular bundles. According to the nucleotype theory, polyploidization increases nuclear volume, which necessitates a proportional expansion of cellular and vacuolar space to maintain the nucleo-cytoplasmic ratio [31]. This expanded physical “sink capacity” in tetraploids creates a dominant physiological pull for photoassimilates [32] and facilitates more efficient partitioning of photoassimilates transported via the vascular system, enabling enhanced starch sequestration within the bulb tissues [33]. Such specialized storage dynamics directly contribute to the biomass accumulation and individual bulblet hypertrophy observed in tetraploids.

At the molecular level, the regeneration and enlargement of in vitro lily bulblets are closely associated with the coordinated regulation of carbohydrate metabolism and hormone signaling. Earlier investigations have shown that the formation and enlargement of storage organs depend on the proper allocation of photosynthates between source and sink tissues [34].

In tetraploid *L. longiflorum*, the rapid enlargement stage (24–36 d) was characterized by the synchronized upregulation of *LlSWEET15* and starch biosynthesis genes (*LlAGPS1* and *LlGBSSI*). This expression pattern aligns with findings in *Lilium brownii* var. *giganteum* [11], *Lycoris radiata* [9] and sweet potato [35], illustrating that the massive translocation of sucrose into sink tissues, coupled with efficient starch biosynthesis and sequestration, not only serves as an energy reservoir for growth but also enhances sink strength, thereby promoting the transport of water and nutrients toward the bulblet and facilitating cell expansion and tissue enlargement [9]. Meanwhile, hormone-responsive genes associated with growth regulation (*LlSAUR32* and *LlMYC2*) were synchronously upregulated during the bulblet enlargement stage in tetraploid lilies. This pattern is consistent with previous observations in the development and enlargement of storage organs such as potato, tomato and *Lycoris radiata* [9,36,37]. Previous studies have shown that hormonal signaling improves the level of cellular plasticity resulting in extra space to store carbohydrates as well as eventually increase the size of the bulblet [38].

In contrast, diploids exhibited an expression pattern predominantly characterized by enhanced sucrose catabolism during in vitro bulblet enlargement. The expression levels of *LlSUS4* and *LlCWIN3* were consistently higher in diploids than in tetraploids and increased progressively with bulblet development, which closely corresponded to the diploid phenotype of continuously producing a greater number of newly formed bulblets in vitro. Similar to this process, during the production of new bulblets, *LiSUS4* has been found to be highly up regulated in *Lycoris radiata* [9], and *LbgCWIN1* has been suggested to be involved in the initiation of in vitro bulb formation in lilies [39]. These observations point that heightened expression levels of genes that encode sucrose synthase (SUS) and cell wall invertase (CWIN) facilitate rapid carbon supply and signaling inputs to support cell division and the formation of new organs.

In summary, our results demonstrate that diploid and tetraploid *L. longiflorum* employ distinct developmental programs, characterized by a division-oriented strategy in diploids versus an expansion-oriented strategy in tetraploids. The synchronized upregulation of starch-biosynthesis genes, coupled with cytological evidence of increased granule density, underscores the superior sink strength and carbon sequestration capacity inherent in tetraploids. Further research involving the dynamic quantification of non-structural carbohydrates will be essential to precisely map these ploidy-dependent carbon allocation patterns.

## 4. Materials and Methods

### 4.1. Plant Materials and Culture Conditions

The diploid *L. longiflorum* bulbs utilized in this study were sourced from the germplasm repository of the Beijing Flower and Wood Group Cultivation Center (Beijing, China; 116°21′9.60″ E, 39°50′24.26″ N). The tetraploid plants (4 L) were generated in our laboratory through somatic chromosome doubling of diploid (2 L) scales. In vitro bulblets with diameters of 0.4–0.6 cm were obtained after long-term subculture. All experimental materials involved in the following experiments were cultured into tissue culture bottles with a height of 9.2 cm and a diameter of 6.7 cm. The culture room was maintained at 24 ± 2 °C with a light intensity of 2500–3500 lx (Conventional fluorescent lamps were used as the light source, providing a photosynthetic photon flux density (PPFD) of approximately 30–50 μmol·m^−2^·s^−1^) and a photoperiod of 14 h/d.

### 4.2. Chromosome Preparation and Ploidy Level Observation Using the Root Tip Squash Method

When the root length of in vitro *L. longiflorum* (both 2 L and 4 L) reached 0.5–1.0 cm, a total of 20 plantlets were randomly selected from 10 culture bottles. The roots were excised, thoroughly rinsed, and pre-treated in ice water for 24 h, followed by fixation in Carnoy’s solution (absolute ethanol: glacial acetic acid = 3:1, *v*/*v*) overnight. The fixed root tips were then macerated in an enzyme mixture containing 1% cellulase and 1% pectinase in a 37 °C water bath. Subsequently, the samples were stained with 2% aceto-carmine and prepared using the squash method. Observations were performed under an optical microscope (Leica, Wetzlar, Germany) to identify and photograph the optimal metaphase spreads.

### 4.3. Adventitious Bud Induction Test of Scales

Outer and middle scales were excised from in vitro–grown diploid and tetraploid *L. longiflorum* plantlets, and explants were placed on the three kinds of culture media described below with the adaxial surface facing upward:

1. To investigate the effects of CPPU (BIORIGIN, Beijing, China) in combination with 6-BA (BIORIGIN, Beijing, China) or NAA (BIORIGIN, Beijing, China) on ad-ventitious shoot induction, a three-factor experimental design was imple-mented using Murashige and Skoog (MS) (PhytoTech, Lenexa, KS, USA) as the basal medium. The experiment comprised seven treatment combinations involving CPPU (0, 0.1 and 0.5 mg/L), 6-BA (0, 0.5 and 1.0 mg/L) and NAA (0, 0.2 mg/L). The specific treatments are shown in Table 1.
2. Effects of glyphosate (Macklin, Shanghai, China) on adventitious shoot induction: Based on the optimal induction and differentiation medium for *L. longiflorum* bulb scales identified in previous studies (MS + 0.5 mg/L 6-BA + 0.2 mg/L NAA) [40], a singal-factor de-sign was employed. The experiment comprised four treatment combinations involving glyphosate (0, 1.0, 2.0, and 3.0 mg/L), The specific treatments are shown in Table 2.
3. Effects of TDZ (BIORIGIN, Beijing, China) on adventitious shoot induction: Based on the medium (1/2 MS + NAA 1.0 mg/L), a singal-factor design was employed. The experiment com-prised four treatment combinations involving TDZ (0, 0.2, 0.5 and 1.0 mg/L). The specific treatments are shown in Table 3.

All differentiation media were supplemented with 30 g/L sucrose (BIORIGIN, Beijing, China) and 7 g/L agar (BIORIGIN, Beijing, China), with the pH adjusted to 5.8–6.0, followed by autoclaving at 121 °C (approx. 108 kPa) for 20 min. Each treatment consisted of three replicates, with 24 scales cultured per replicate. The differentiation rate was monitored daily, and growth was documented photographically. After 35 days, the shoot induction frequency and differentiation coefficient were calculated.

Shoot induction frequency = number of explants producing adventitious shoots/total number of cultured explants. Values are expressed as ratio values.

Differentiation coefficient = total number of adventitious shoots (defined as shoots longer than 2 mm)/number of explants producing adventitious shoots.

### 4.4. Experimental Design for In Vitro Bulblet Enlargement

In the in vitro bulblet enlargement experiment, the fresh weight of both ploidy levels of *L. longiflorum* bulblets was measured individually using an electronic balance prior to cultivation. Bulblets weighing between 0.10–0.20 g were chosen and subsequently cultured in various media treatments. Each treatment consisted of three replicates, with 10 bulblets cultured per replicate, for a total of 30 bulblets per treatment. As documented in the literature and supported by our previous work, an L9 (3^4^) orthogonal experimental design for both ploidy levels of *L. longiflorum* was employed to evaluate the effects of four factors—sucrose, IBA (BIORIGIN, Beijing, China), 6-BA, and CPPU—and their different concentrations on in vitro bulblet enlargement in two ploidy levels of *L. longiflorum*. The experiment comprised nine treatment combinations involving sucrose (30, 60, and 90 g/L), IBA (0.5, 1.0, and 2.0 mg/L), 6-BA (0, 0.1, and 0.5 mg/L) and CPPU (0, 0.1, and 0.2 mg/L). The detailed experimental design is shown in Table 6. After 60 days of culture, bulblet fresh weight, diameter, and the bulblet enlargement coefficient were recorded.

Bulblet enlargement coefficient = fresh weight of bulblets after culture/initial fresh weight of bulblets (The bulblet enlargement coefficient for each replicate was calculated as the average of the bulblet enlargement coefficients of the individual bulblets within that replicate)

In this experiment, Murashige and Skoog (MS) medium was used as the basal medium and solidified with 7 g/L agar. The pH was precisely adjusted to 5.80–6.00 by adding 1 mol/L potassium hydroxide or hydrochloric acid solution, followed by autoclaving at 121 °C (approx. 108 kPa) for 20 min.

### 4.5. Dynamic Observation of Bulblet Enlargement and Associated Gene Expression Analysis

#### 4.5.1. Observation of Bulblet Enlargement Dynamics

Prior to culture, individual bulblets of both diploid and tetraploid *L. longiflorum* were weighed using an electronic balance. Only those weighing between 0.10 and 0.20 g were selected and then cultured under optimized medium treatments (diploid: MS + 60 g/L sucrose + 2.0 mg/L IBA; tetraploid: MS + 60 g/L sucrose + 0.5 mg/L IBA + 0.1 mg/L 6-BA + 0.1 mg/L CPPU). For each ploidy and each time point, three replicates were prepared, each containing 10 bulblets, giving a total of 30 bulblets per treatment. Bulblets were photographed at six time points (0, 12, 24, 36, 48, and 60 days of culture). The fresh weight, bulblet enlargement coefficient, and the number of daughter bulblets were recorded at each time point.

Bulblet enlargement coefficient (see Section 4.4 for further details.)

The number of daughter bulblets = total number of daughter bulblets per replicate (defined as newly formed bulblets differentiated from the original mother bulblet, measuring >2 mm in diameter with distinct scale structures.)/the number of cultured mother bulblets in that replicate.

#### 4.5.2. Cytological Observation of In Vitro Bulblet Scale Structure and Starch Accumulation

Small scales were excised from in vitro–grown diploid and tetraploid *L. longiflorum* bulblets cultured for 60 days. The middle regions of the scales were sectioned transversely and longitudinally, fixed in FAA solution, and subsequently dehydrated through a graded ethanol series and cleared with xylene. The samples were then embedded in paraffin using standard procedures [41]. Sections of 12 μm thickness were prepared using a rotary microtome (Leica RM2245, Wetzlar, Germany). Finally, the sections were stained with periodic acid–Schiff (PAS) reagent for polysaccharide detection and observed [42]. Final observations and imaging were performed under a light microscope (Leica DM500, Grenchen, Switzerland).

#### 4.5.3. Expression of Genes Associated with the Development of In Vitro Bulblets

Bulblet samples were harvested at 0, 12, 24, 36, 48, and 60 days of cultivation. Total RNA was extracted using the RNAprep Pure Polysaccharide and Polyphenol Total RNA Extraction Kit (DP441, Tiangen, Beijing, China) following the manufacturer’s protocols. The integrity and concentration of the extracted RNA were verified via agarose gel electrophoresis and spectrophotometry, respectively. Subsequently, complementary DNA (cDNA) was synthesized using the ReverTra Ace qPCR RT Master Mix Kit (FSQ-301, Toyobo, Osaka, Japan). The candidate gene sequences related to carbohydrate metabolism and phytohormonal signaling were retrieved from an in-house transcriptome database of *L. longiflorum* (unpublished data) previously established in our laboratory. To ensure robust quantification across different ploidy levels, the lily *Actin* gene (GenBank accession No. JX826390) was utilized as the internal reference after validating its expression stability [43]. Gene-specific primers (Table 7) were designed using Primer Premier 6.0 (Premier Biosoft International, Palo Alto, CA, USA), targeting conserved regions to ensure high compatibility for both diploid and tetraploid genotypes. All primers were synthesized by Sangon Biotech (Shanghai, China). The specificity of the primers was verified by NCBI Primer-BLAST and confirmed by melting curve analysis after the PCR reaction. Quantitative PCR was performed using the SYBR Green Pro Taq HS premix kit (AG11701, AG, Changsha, China) on a real-time PCR system (Bio-Rad CFX Duet, Hercules, CA, USA). The total volume of the reaction system was 20 μL, consisting of 10 μL of 2 × SYBR Green *Pro Taq* HS *Premix*, 0.5 μL each of forward and reverse primers (10 μmol/L), 2 μL of cDNA template, and 7 μL of ddH2O. The amplification program was set as follows: 95 °C for 30 s; followed by 40 cycles of 95 °C for 5 s and 60 °C for 30 s. All reactions were conducted in triplicate and relative gene expression levels were calculated using the 2^−ΔΔCT^ method.

### 4.6. Data Analysis

Statistical analysis was performed using SPSS 24.0 software. Significant differences and multiple comparisons between treatments were determined by Duncan’s multiple range test with the significance level set at *p* < 0.05. Data processing and figure preparation were conducted using Microsoft Excel 2016 and GraphPad Prism 9.5.

## 5. Conclusions

This study systematically elucidated the factors governing the in vitro regeneration and bulblet enlargement of diploid and tetraploid *Lilium longiflorum*. The results demonstrated that both CPPU and glyphosate enhance adventitious bud differentiation to varying degrees. Notably, the addition of TDZ to the culture medium induced a shift in the regenerative pathway, transitioning from direct organogenesis to a callus-mediated indirect regeneration route. Regarding differentiation efficiency, diploid *L. longiflorum* was most productive via direct adventitious bud formation, with the optimal induction medium identified as MS + 30 g/L sucrose + 0.5 mg/L 6-BA + 0.2 mg/L NAA + 1.0 mg/L glyphosate. Conversely, tetraploid L. longiflorum exhibited higher efficiency through the callus-mediated pathway, for which the ideal medium was 1/2 MS + 30 g/L sucrose + 1.0 mg/L NAA + 0.2 mg/L TDZ.

During the bulblet enlargement stage, diploids showed greater sensitivity to IBA, reaching optimal growth on MS + 60 g/L sucrose + 2.0 mg/L IBA. In contrast, tetraploids were highly responsive to sucrose concentration, with the most effective medium being MS + 60 g/L sucrose + 0.5 mg/L IBA + 0.1 mg/L 6-BA + 0.1 mg/L CPPU. Physiological observations revealed a significant divergence in developmental strategies: tetraploids prioritized the volumetric hypertrophy of individual bulblets, whereas diploids favored the proliferation of basal daughter bulblets.

At the molecular level, this strategic divergence is driven by distinct gene expression profiles. In tetraploids, the synchronized upregulation of starch biosynthesis genes (*LlAGPS1*, *LlGBSSI*), the sucrose transporter (*LlSWEET15*), and hormone-responsive regulators (*LlMYC2*, *LlSAUR32*) coincided with the rapid enlargement phase (days 24–36), indicating their pivotal roles in promoting bulblet hypertrophy. Meanwhile, the elevated expression of *LlSUS4* and *LlCWIN3* in diploids is likely associated with carbon allocation during bulblet proliferation. This paper has determined the best culture conditions to use to promote bulblet enlargement and scale differentiation of both diploid and tetraploid L. longiflorum, a technical contribution to the development of high-throughput production systems. Physiological and gene expression analyses during bulblet enlargement provide a theoretical basis for future research into the mechanisms governing bulblet enlargement in tissue-cultured lilies of different ploidies.

## Figures and Tables

**Table 1:** Effects of CPPU combined with 6-BA or NAA on scale-induced culture of two ploidy *L. longiflorum*.

Plant Growth Regulators			4 L				2 L			
CPPU mg/L	6-BA mg/L	NAA mg/L	Differentiation Pathway	Germination Time/d	Shoot Induction Frequency	Differentiation Coefficient	Differentiation Pathway	Germination Time/d	Shoot Induction Frequency	Differentiation Coefficient
0	0.5	0.2	Adventitious shoots	20–40	0.93 ± 0.13	1.90 ± 0.31 ^bcd^	Adventitious shoots	25–40	1.0	2.51 ± 0.32 ^a^
0.1	0.5	0	Adventitious shoots	24–40	0.96 ± 0.06	2.48 ± 0.14 ^b^	Adventitious shoots	20–40	0.98 ± 0.05	1.90 ± 0.22 ^bc^
0.1	1.0	0	Adventitious shoots	18–40	1.0	2.15 ± 0.22 ^cd^	Adventitious shoots	16–40	1.0	1.87 ± 0.18 ^bc^
0.5	0.5	0	Adventitious shoots	18–40	1.0	2.30 ± 0.13 ^bc^	Adventitious shoots	16–40	0.95 ± 0.11	1.75 ± 0.23 ^c^
0.5	1.0	0	Adventitious shoots	24–40	1.0	1.82 ± 0.10 ^d^	Adventitious shoots	20–40	1.0	1.71 ± 0.15 ^c^
0.1	0	0.2	Adventitious shoots	14–35	1.0	3.45 ± 0.44 ^a^	Adventitious shoots	16–40	1.0	2.15 ± 0.13 ^ab^
0.5	0	0.2	Adventitious shoots	18–35	0.96 ± 0.06	3.41 ± 0.17 ^a^	Adventitious shoots	20–40	1.0	2.56 ± 0.54 ^a^

Note: Data are average ± standard error. Different lowercase letters in the same column represent the difference significance of data at the *p* < 0.05 level.

**Table 2:** Effects of glyphosate on scale-induced culture of two ploidy *L. longiflorum*.

Glyphosate mg/L	4 L				2 L			
	Differentiation Pathway	Germination Time/d	Shoot Induction Frequency	Differentiation Coefficient	Differentiation Pathway	Germination Time/d	Shoot Induction Frequency	Differentiation Coefficient
1.0	Adventitious shoots	30–45	0.89 ± 0.19	1.83 ± 0.17 ^c^	Adventitious shoots	25–50	1.0	3.64 ± 0.30 ^a^
2.0	Adventitious shoots	25–40	1.0	2.26 ± 0.06 ^b^	Adventitious shoots	30–55	1.0	3.08 ± 0.19 ^b^
3.0	Adventitious shoots	25–40	0.93 ± 0.13	2.88 ± 0.25 ^a^	Adventitious shoots	35–60	0.96 ± 0.06	3.26 ± 0.36 ^b^

Note: Data are average ± standard error. Different lowercase letters in the same column represent the difference significance of data at the *p* < 0.05 level.

**Table 3:** Effects of TDZ on scale-induced culture of two ploidy *L. longiflorum*.

	4 L				2 L			
TDZ mg/L	Differentiation Pathway	Germination Time/d	Shoot Induction Frequency	Differentiation Coefficient	Differentiation Pathway	Germination Time/d	Shoot Induction Frequency	Differentiation Coefficient
0 (control)	Adventitious shoots	24–45	1.0	2.81 ± 0.45 ^c^	Adventitious shoots	20–40	0.92 ± 0.06	1.41 ± 0.38 ^b^
0.2	callus	18–40	1.0	4.15 ± 0.61 ^a^	callus	35–50	0.93 ± 0.15	2.09 ± 0.25 ^a^
0.5	callus	20–40	1.0	3.41 ± 0.45 ^b^	callus	35–50	0.98 ± 0.05	1.42 ± 0.25 ^b^
1.0	callus	20–40	1.0	3.48 ± 0.17 ^b^	callus	35–50	0.92 ± 0.11	1.83 ± 0.20 ^ab^

Note: Data are average ± standard error. Different lowercase letters in the same column represent the difference significance of data at the *p* < 0.05 level.

**Table 4:** The results of L_9_ (3^4^) orthogonal test on bulblet enlargement of diploid and tetraploid *L. longiflorum*.

Code	Sucrose g/L	IBA mg/L	6-BA mg/L	CPPU mg/L	4 L		2 L	
					Bulblet Diamete (mm)	Enlargement Coefficient	Bulblet Diameter/mm	Enlargement Coefficient
1	30	0.5	0	0	9.41 ± 0.96 ^bcd^	3.13 ± 0.68 ^c^	7.22 ± 0.69 ^cd^	2.31 ± 0.49 ^de^
2	30	1.0	0.1	0.1	9.96 ± 0.65 ^ab^	3.88 ± 0.78 ^ab^	6.86 ± 0.59 ^d^	2.46 ± 0.50 ^ce^
3	30	2.0	0.5	0.2	9.28 ± 0.86 ^cd^	2.94 ± 0.62 ^c^	7.87 ± 0.64 ^ab^	2.96 ± 0.37 ^ab^
4	60	0.5	0.1	0.2	10.23 ± 0.69 ^a^	4.08 ± 0.97 ^a^	7.14 ± 0.80 ^cd^	2.58 ± 0.51 ^be^
5	60	1.0	0.5	0	9.63 ± 1.09 ^bc^	2.88 ± 0.76 ^c^	6.99 ± 0.94 ^cd^	2.71 ± 0.45 ^bcd^
6	60	2.0	0	0.1	9.53 ± 0.85 ^bcd^	3.20 ± 0.76 ^bc^	8.11 ± 0.46 ^a^	3.24 ± 0.42 ^a^
7	90	0.5	0.5	0.1	8.33 ± 0.93 ^e^	2.66 ± 0.87 ^cd^	8.21 ± 0.48 ^a^	2.39 ± 0.44 ^e^
8	90	1.0	0	0.2	9.00 ± 0.97 ^d^	2.10 ± 0.61 ^d^	7.47 ± 0.78 ^bc^	2.55 ± 0.36 ^be^
9	90	2.0	0.1	0	8.32 ± 0.27 ^e^	1.92 ± 0.14 ^d^	7.87 ± 0.95 ^ab^	2.90 ± 0.41 ^abc^

Note: Data are average ± standard error. Different lowercase letters in the same column represent the difference significance of data at the *p* < 0.05 level.

**Table 5:** Analysis of variance (ANOVA) of the effects of sucrose, IBA, 6-BA, and CPPU on bulblet enlargement in 2 L and 4 L.

	Factor	SS	*df*	MS	F	*p*
4 L	sucrose	45.85	2	22.93	43.34	<0.001 **
	IBA	10.59	2	5.30	10.01	<0.001 **
	6-BA	8.21	2	4.11	7.76	<0.001 **
	CPPU	7.64	2	3.81	7.22	0.001 **
	error	80.93	153	0.53		
	total	1589.15	162			
2 L	sucrose	2.695	2	1.347	6.490	0.002 **
	IBA	7.371	2	3.685	17.751	0.001 **
	6-BA	0.132	2	0.066	0.317	0.729
	CPPU	0.152	2	0.076	0.366	0.694
	error	31.764	153	0.208		
	total	1193.308	162			

Note: ** represent extremely significant at *p* < 0.01 level.

**Table 6:** Different media for the bulblet enlargement of diploid and tetraploid *L. longiflorum*.

Code	Sucrose g/L	Plant Growth Regulator (mg/L)		
		IBA mg/L	6-BA mg/L	CPPU mg/L
1	30	0.5	0	0
2	30	1.0	0.1	0.1
3	30	2.0	0.5	0.2
4	60	0.5	0.1	0.2
5	60	1.0	0.5	0
6	60	2.0	0	0.1
7	90	0.5	0.5	0.1
8	90	1.0	0	0.2
9	90	2.0	0.1	0

**Table 7:** Primers used for Real-time quantitative PCR analysis.

Gene	Forward Primer Sequence (5′→3′)	Reverse Primer Sequence (5′→3′)
*LlGBSS1*	AGCCGTGTGGGCTTATTCAA	TAACAGTCGCCACCACATCC
*LlAGPS1*	CGAAGAAGAGAGCCAAGCCA	CCATGTTGCTCGCATAAGCC
*LlMYC2*	CGGGGCAGATGTGATGCTTA	TCGACATCTTCACCGTCAGC
*LlSWEET15*	TTGGATGTGTGCTTGTTCGG	TCCTCGTCCGAATGACAAGC
*LlSAUR32*	TGGGTCACAACTCACAGAACC	GGGTGGGTAAGGGACATCAC
*LlSUS4*	GCCCACCGCAATGATCTAGT	AGAGCGTCGAAGAAAGGTCC
*LlCWIN3*	CCACCATCCTCCCTGGCAAC	CCGTCGGATCCCATCCAAGC
*Actin* (reference gene)	CCCATTGAGCACGGCATTGTC	GGATTGAGAGGAGCTTCGGTGAGA

## Data Availability

Data are contained within the article.
